# Usability and accuracy of two different aortic annulus sizing software programs in patients undergoing transcatheter aortic valve replacement

**DOI:** 10.1186/s44348-024-00002-9

**Published:** 2024-05-29

**Authors:** Johannes Spanke, Jonathan Nübel, Frank Hölschermann, Grit Tambor, Claudia Kiessling, Hidehiro Kaneko, Anja Haase-Fielitz, Christian Butter

**Affiliations:** 1 Department of Cardiology, Heart Centre Brandenburg Bernau & Faculty of Health Sciences Brandenburg, Brandenburg Medical School (MHB) Theodor Fontane, Ladeburger Straße 17, Bernau bei Berlin, 16321 Germany; 2 Personal and Interpersonal Development in Health Care Education, University Witten/Herdecke, Witten, Germany; 3 Department of Cardiovascular Medicine, The University of Tokyo Hospital, Tokyo, Japan; 4 Institute of Social Medicine and Health Care Systems Research, Otto Von Guericke University Magdeburg, Magdeburg, Germany

**Keywords:** Transcatheter aortic valve replacement, Cardiac imaging techniques, Software, Usability testing, Multislice computed tomography

## Abstract

**Background:**

Semi-automated software is essential for planning and prosthesis selection prior transcatheter aortic valve replacement (TAVR). Reliable data on the usability of software programs for planning a TAVR is missing. The aim of this study was to compare software programs ‘Valve Assist 2’ (GE Healthcare) and 3mensio ‘Structural Heart’ (Pie Medical Imaging) regarding usability and accuracy of prosthesis size selection in program-inexperienced users.

**Methods:**

Thirty-one participants (*n* = 31) were recruited and divided into program-inexperienced users (beginners) (*n* = 22) and experts (*n* = 9). After software training, beginners evaluated 3 patient cases in 129 measurements (*n* = 129) using either Valve Assist 2 (*n* = 11) or Structural Heart (*n* = 11) on 2 test days (T1, T2). System Usability Scale (SUS) and ISONORM 9241/110-S (ISONORM) questionnaire were used after the test. The valve size selected by each beginner was compared with the valve size selected from expert group.

**Results:**

Valve Assist 2 had higher SUS Score: median 78.75 (25th, 75th percentile: 67.50, 85.00) compared to Structural Heart: median 65.00 (25th, 75th percentile: 47.50, 73.75), (*p* < 0,001, *r* = 0.557). Also, Valve Assist 2 showed a higher ISONORM score: median 1.05 (25th, 75th percentile: − 0.19, 1.71) compared to Structural Heart with a median 0.05 (25th, 75th percentile: − 0.49, 0.13), (*p* = 0.036, *r* = 0.454). Correctly selected valve sizes were stable over time using Valve Assist 2: 72.73% to 69.70% compared to Structural Heart program: 93.94% to 40% (χ^2^ (1) = 21.10, *p* < 0.001, φ = 0.579).

**Conclusion:**

The study shows significant better usability scores for Valve Assist 2 compared to 3mensio Structural Heart in program-inexperienced users.

## Introduction

Aortic stenosis (AS) is among the most common valvular heart diseases [1]. The prevalence of AS increases with age and is especially high in the elderly [2], affecting 8% of patients aged 85 years or older [3].

Transcatheter aortic valve replacement (TAVR) is a minimally invasive therapy for patients with symptomatic AS and commonly used. To achieve the best interventional results, annular sizing is crucial for prothesis selection prior the procedure [4]. Pre- procedural sizing requires precise knowledge of the anatomic dimensions and physical characteristics of the aortic valve, annulus, and aortic root [4]. Rigorous assessment of device landing zone is an essential component of risk stratification and procedural planning in patients undergoing TAVR.

For preprocedural sizing, multislice computed tomography (MSCT) image data is most accurate and clinically regular used [5–7]. Semi-automatic software supports sizing, as it is more structured and time saving than manual evaluation [8–11]. Various commercial software programs are used for sizing and planning a TAVR  [12, 13].

Anulus measurement and resulting choice of prosthesis’ size is particularly crucial for patient’s outcome [14]. While oversizing might result in life threatening annular rupture, undersizing can lead to post-procedural paravalvular leakage [14, 15].

Considering these complications and the fact that the usability of a medical device has a direct impact on patient’s outcome [16], usability differences between 3mensio “Structural Heart” (Pie Medical Imaging, Maastricht, The Netherlands) and “Valve Assist 2” (GE Healthcare, Chicago, IL, USA) are of interest. Methods of usability research include standardized questionnaires [17, 18] such as the System Usability Scale (SUS) and ISONORM 9241/110-S (ISONORM), which are valid methods [19, 20] to identify usability problems.

However, reliable data on the usability of software programs for preprocedural sizing and planning a TAVR is missing. In this prospective study, we assessed usability and accuracy of 2 different aortic annulus sizing software programs in program-inexperienced users.

## Methods

### Study design

An experimental prospective randomized controlled 2-arm study was designed to compare usability of Structural Heart and Valve Assist 2.

The study was conducted from November 29 to December 21, 2018, at the Department of Cardiology, Heart Centre Brandenburg Bernau & Faculty of Health Sciences Brandenburg, Brandenburg Medical School (MHB) Theodor Fontane, in Bernau, Germany.

### Participants

**Fig. 1:**
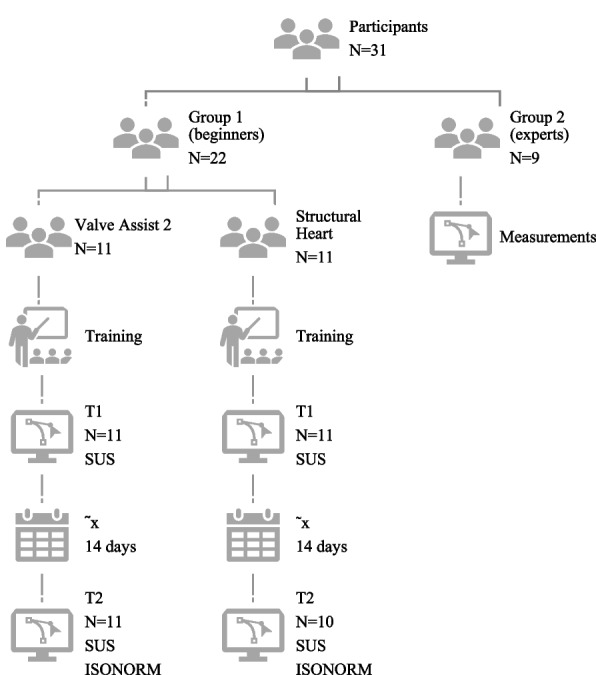
Flow through the study. ISONORM: ISONORM 9241/110-S, SUS: System Usability Scale, T1: test day 1, T2: test day 2

Thirty-one participants (*n* = 31) were recruited for our study (Fig. 1). Participants were divided into 2 groups:

Group 1: program inexperienced users (beginners) (*n* = 22) and Group 2: reference group/experts (*n* = 9). Inclusion criteria for Group 1 were software inexperienced (a) cardiology residents, (b) medical students after completing German federal licensing examination step 1, and (c) informed consent to participation. Exclusion criteria were (a) clinical experience with software Structural Heart or Valve Assistant 2 and (b) lack of informed consent. Group 2 included (a) interventional cardiologists using the software when planning a TAVR (*n* = 4), (b) trained specialists from the manufacturer (*n* = 1), and (c) radiologists (*n* = 4). Exclusion criterion was lack of informed consent. Group 1 were randomly assigned to either Structural Heart or Valve Assist 2 (Fig. 1).

One student from the Structural Heart group did not show up for test day 2 (T2).

### Tools and tests

Three anonymized patient MSCT images from the Heart Centre Brandenburg Bernau were used. Selection criteria were the indication for TAVR and a minimum image quality.

A German version of SUS [21] and ISONORM [22, 23] questionnaire were used, the latter being a short form and further development [24] of Prümper’s ISONORM 9241/10 [25] to evaluate usability. The technology-independent SUS consists of a questionnaire with ten items, each with a 5-point answer option on a Likert scale, ranging from strongly agree to strongly disagree [19]. ISONORM consists of a questionnaire with 21 items, each with a 7-point answer option on a Likert scale, ranging from (+ + +) positive to (− − −) negative [24].

ISONORM evaluates the 7 dialogue principles of Ergonomics of human-system interaction (ISO 9241-110:2006) according to International Organization for Standardization (ISO).

### Training procedures

Group 1 received a 30-min training for one of the software programs under standardized conditions: (a) separated room, (b) laptop with the respective software program, (c) video projector with screen, and (d) seats facing the screen. The training was led by user specialists with experience for the relevant program. Questions were allowed but no written records.

### Test procedure

**Fig. 2:**
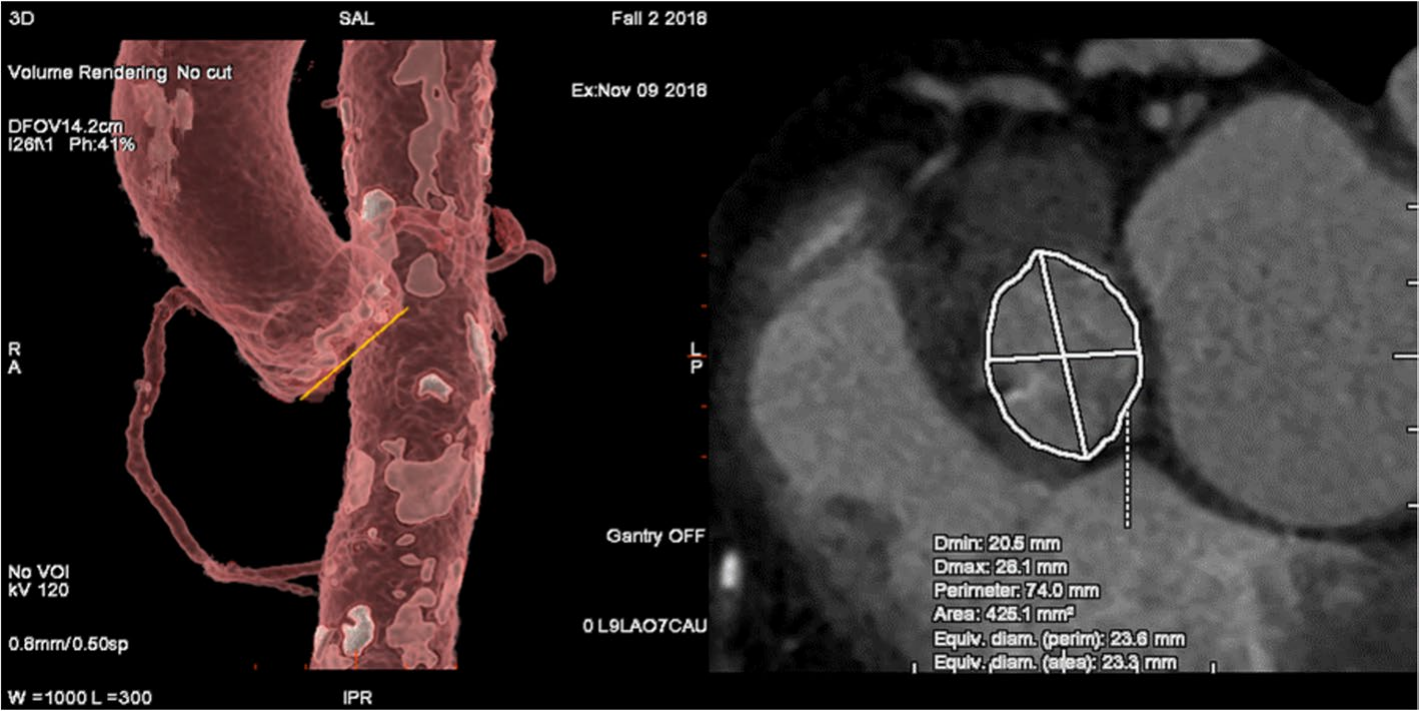
Measurements with Valve Assist 2

For Group 1 experimental phase was divided into 2 test days T1 and T2. There was a minimum of 13 and a maximum of 22 (median: 14; 25th, 75th percentile: 14.00, 14.00) training free days between T1 and T2. Beginners evaluated the parameters perimeter (mm), area (mm^2^), right coronary artery height measurement (annulus to right coronary ostia; mm), left coronary artery height measurement (annulus to left coronary ostia; mm) and implant angle (aortic annulus angulations for fluoroscopy) (Fig. 2).

The duration of each measurement in minutes (time on task) was recorded. If beginners were at a loss, they could ask the expert for a hint. The numbers of questions necessary to perform measurement was recorded. For comparison, Group 2 measured the same parameters and patient samples. After T1 SUS and after T2 SUS [21] and ISONORM [22] were recorded.

### Statistics

Kolmogorov-Smirnov test and Shapiro-Wilk test were used to test for normal distribution. Non-normally distributed and ordinal data were reported as median with 25th to 75th percentiles and compared using Mann-Whitney U test (independent samples) or Wilcoxon- Test (dependent samples). Normally distributed data were reported as mean with standard deviation and compared using t-test. For nominal data chi-square test was used. For nominal data with less than 5 expected cell counts Fisher-Freeman-Halton exact test was used. According to Cohen [26] effect size was reported as d for t-test, as r for Whitney U test and as φ for χ^2^ test.

SUS score was calculated and ranged between 0–100 [27]. SUS score data of T1 and T2 were summarized. Following the approach of [28], the SUS score was linked with US academic grading from A–F. For comparison, the median for ISONORM and each dialogue principles were used and ranged between −3 to +3 [17].

If area (mm^2^) measurement of beginners in Group 1 and mean of the experts in Group 2 were within the same limit range [29] according to the SAPIEN 3 transcatheter valve (Edwards Lifesciences, Irvine, CA, USA), the measurement was considered as correct. Intraclass correlation coefficient (ICC) was used in the interrater reliability analyses of the expert group.

Data were anonymized following the principles of the German Psychological Society [30]. An alpha level of 0.05 was set to test for significance. Statistical analysis was performed using IBM SPSS Statistics 25 (IBM, Armonk, NY, USA).

## Results

### SUS

**Fig. 3:**
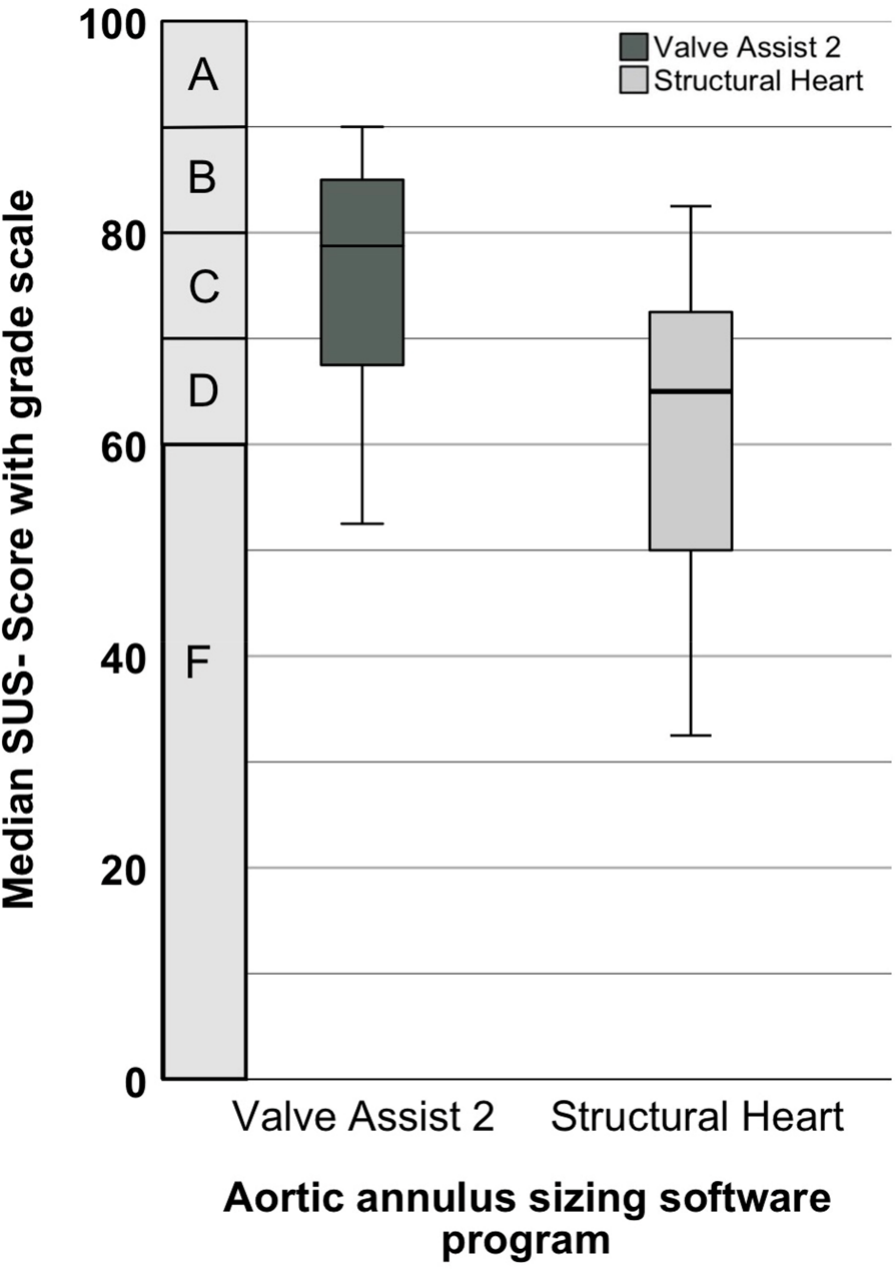
Boxplots for SUS score plotted on the academic/school grade scale separated by an aortic ring sizing software program. SUS: System Usability Scale

SUS score was higher in Valve Assist 2 compared to Structural Heart (median: 78.75 [25th, 75th percentile: 67.50, 85.00] vs. median: 65.00 [25th, 75th percentile: 47.50, 73.75], *p* < 0,001, *r* = 0.557) (Fig. 3). There was no difference in SUS Score between T1 and T2 (Valve Assist 2: *p* = 0.837, Structural Heart: *p* = 0.066).

### ISONORM

**Fig. 4:**
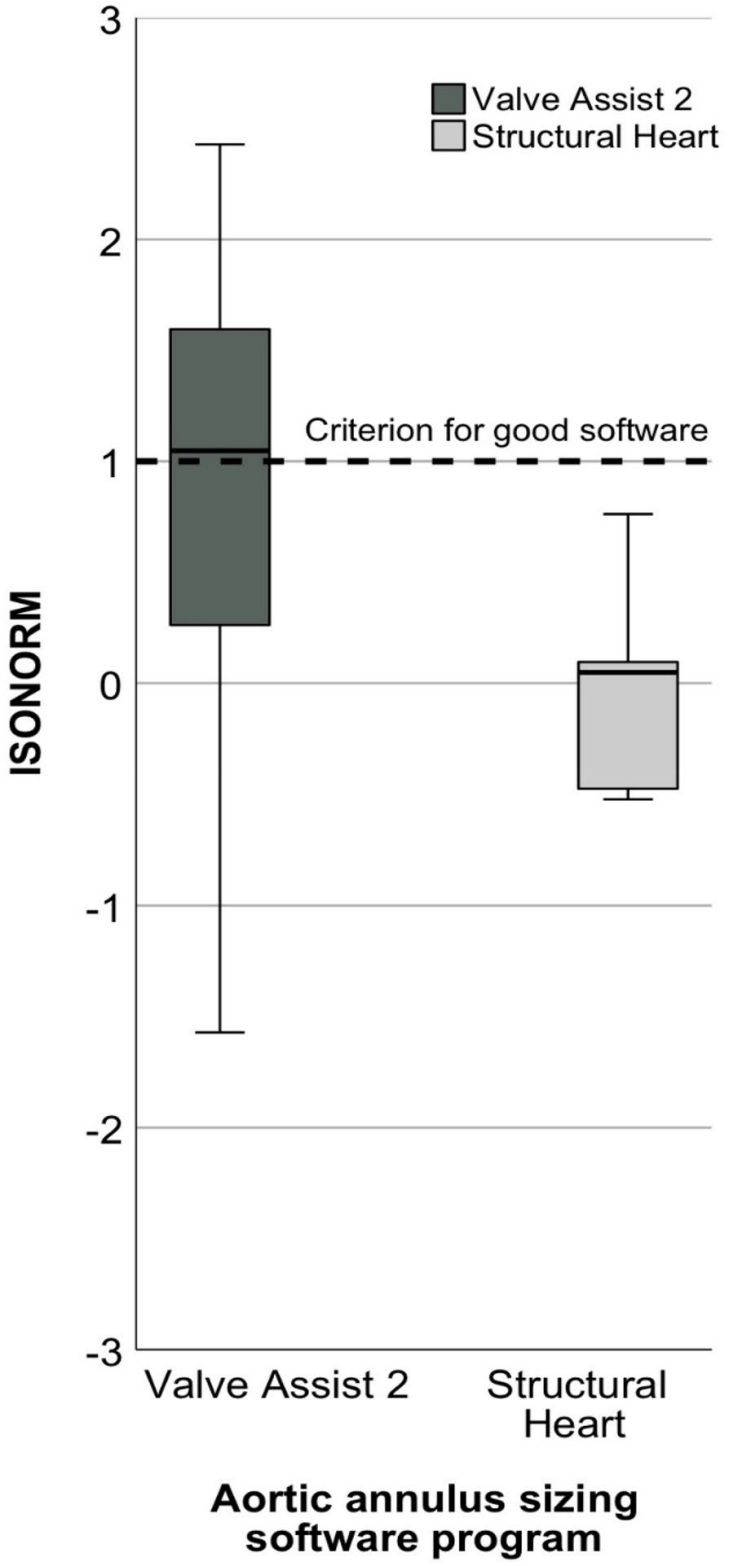
Boxplots for ISONORM with plotted criterion at +1 for good software separated by aortic ring sizing software program. ISONORM: ISONORM 9241/110-S

Also, Valve Assist 2 showed higher ISONORM score compared to Structural Heart (Fig. 4, *p* = 0.036, *r* = 0.454), suitability for learning (*p* = 0.024, *r* = 0.486), suitability for individualization (*p* = 0.020, *r* = 0.524), and self-descriptiveness (*p* = 0.004, *r* = 0.617). No significant differences were found for suitability for the task, conformity with user expectations, controllability, and error tolerance (Table 1).

**Table 1:** ISONORM, time on task and questions for Valve Assist 2 and Structural Heart

Variables	Valve Assist 2 (*n* = 11)	Structural Heart (*n* = 11)	*P*-value	*r*
ISONORM	1.05 (−0.19, 1.71)	0.05 (−0.49, 0.13)	0.036	0.454
Suitability for the task	2.00 (0.67, 2.67)	1.17 (0.67, 1.42)	0.197	
Self-descriptiveness	0.33 (−0.67, 1.67)	−1.67 (−2.00, −0.75)	0.004	0.617
Controllability	0.67 (−1.00, 1.67)	0.50 (−0.75, 1.33)	0.605	
Conformity with user expectations	1.67 (0.00, 2.33)	0.33 (0.00, 0.67)	0.051	
Error tolerance	0.00 (−1.00, 2.00)	−0.50 (−1.00, 0.08)	0.282	
Suitability for individualization	0.33 (0.00, 2.00)	0.00 (−0.33, 0.00)	0.020	0.524
Suitability for learning	2.00 (0.67, 2.33)	0.00 (−0.42, 0.58)	0.024	0.486
Time on task in minutes T1	14.72 (11.57, 20.42)	12.42 (12.08, 15.52)	0.652	
Time on task in minutes T2	11.32 (9.73, 14.63)	16.50 (13.92, 23.40)	0.002	0.645
Number of questions to experts T1	6.00 (5.00, 7.00)	10.00 (7.00, 11.00)	0.040	0.437
Number of questions to experts T2	0.00 (0.00, 0.00)	1.00 (0.00, 2.50)	0.020	0.636

Values are presented as median (range) or number (%)

*ISONORM* ISONORM 9241/110-S, *T1* Test day 1, *T2* Test day 2

### Questions needed for measurements and time on task

The numbers of questions necessary to perform measurements was lower with Valve Assist 2 than with Structural Heart in T1 (*p* = 0.040, *r* = 0.437) and T2 (*p* = 0.020, *r* = 0.636) (Table 1).

On the second test day, measurements with Valve Assist 2 were 5.18 min faster (*p* = 0.002, *r* = 0.645) than with Structural Heart (Table 1).

### Correctly selected valves sizes

In Group 1, 129 valve sizing measurements were made with both programs.

**Fig. 5:**
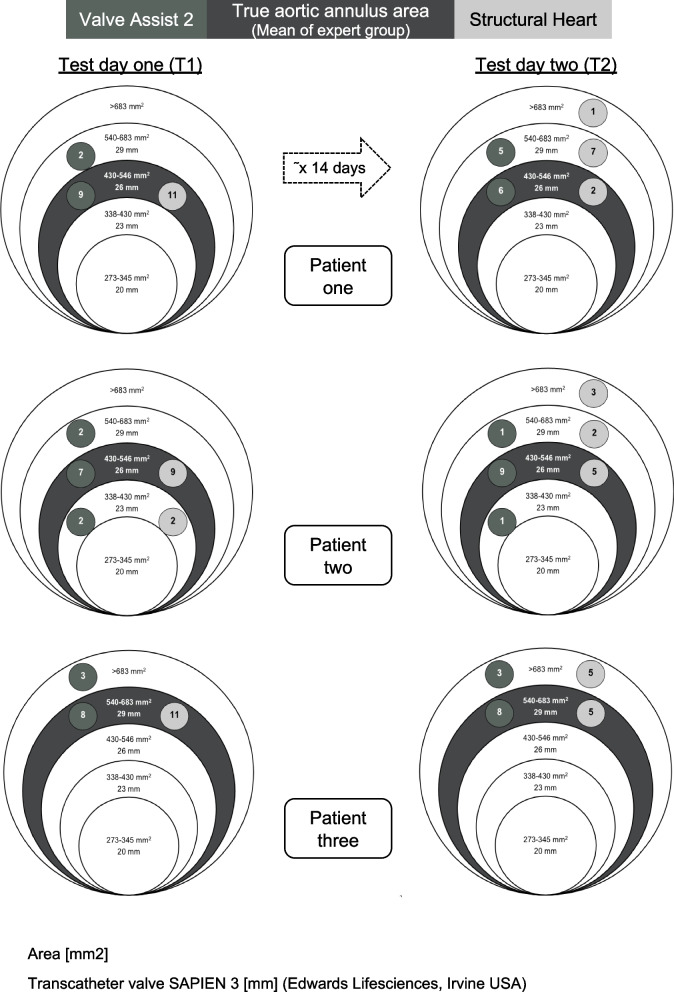
Results of Group 1: area measurements. Comparison of selected valves sizes of 129 measurements from Group 1 (beginners) with the true aortic annulus area (mean of Group 2; experts) considered separately for each patient, test day and the respective software program. T1: test day 1, T2: test day 2

Beginners using Valve Assist 2 chose the correct heart valve in 72.73 percent in T1 and in 69.7 percent in T2 (Fig. 5). Structural Heart achieved a higher percentage with 93.94 percent in T1 and a lower percentage in T2 with 40 percent (Fig. 5). A χ^2^ test showed a difference between software program and correct measured valve size (T1: χ^2^ (1) = 5.35, *p* = 0.021, φ = 0.285, T2: χ^2^ (1) = 5.61, *p* = 0.018, φ = 0.298). With Valve Assist 2 there was no difference between selected correct valve sizes in T1 and T2 (χ^2^ (1) = 0.07, *p* = 0.786). In contrast, there was a difference for Structural Heart between T1 and T2 (χ^2^ (1) = 21.10, *p* < 0.001, φ = 0.579). There was no association between duration of the training-free days between T1 and T2 and correct measured valve size in T2 (*p* = 0.250).

### Results expert

The average measure ICC was 0.981 with a 95% confidence interval from 0.919 to 1 (*F*(2,16) = 53.200, *p* < 0.001) (Table 2).

**Table 2:** Results Group 2/experts: area measurements

Measurements MSCT images Group 2/experts	Patient 1 (*n* = 9)	Patient 2 (*n* = 9)	Patient 3 (*n* = 9)
Area (mm^2^)	488.76 ± 17.58	432.51 ± 18.90	606.80 ± 47.47

Values are presented as mean ± standard deviation

*MSCT* Multislice computed tomography

### Baseline characteristics

Table 3 shows the baseline characteristics of the participants in Group 1 enrolled in the study.

**Table 3:** Baseline characteristics: Group 1

Baseline variable	Total (*n* = 22)	Valve Assist 2 (*n* = 11)	Structural Heart (*n* = 11)	*P*-value
Age (years)	28.00 (24.50, 32.00)	28.00 (24.00, 32.00)	28.00 (26.00, 31.25)	0.973
Age students	24.50 (23.00, 27.25)	24.50 (23.75, 27.25)	25.00 (21.50, 27.75)	0.762
Age doctors	32.00 (29.00, 32.00)	32.00 (30.50, 33.50)	30.00 (28.00, 33.00)	0.329
Female	11 (50)	7 (63.6)	4 (36.4)	
Students	11 (50)	6 (54.5)	5 (45.5)	
Doctors	11 (50)	5 (45.5)	6 (54.5)	
Doctors’ year of training qualifying as a specialist	5.00 (2.00, 6.00)	5.00 (4.00, 7.00)	3.00 (2.00, 5.25)	0.126
Training free days between T1 and T2	14.00 (14.00, 14.00)	14.00 (14.00, 14.00)	14.00 (13.00, 14.00)	0.085

Values are presented as median (range) or number (%)

*T1* Test day 1, *T2* Test day 2

## Discussion

The present study shows that Valve Assist 2 and 3mensio Structural Heart differ in usability. Based on a comparison of usability tests, the findings indicate higher usability results for Valve Assist 2 than Structural Heart.

Two main results underline that there is an advantage for Valve Assist 2 in usability. First, Valve Assist 2 achieves significantly better/higher SUS scores than Structural Heart. While Valve Assist is above the 50th percentile of the known average SUS score (which is at 68) [18], Structural Heart is below. To understand the meaning of SUS scores, we represent SUS score in academic/school grades [28].

In the grades, Valve Assist 2 received a C and Structural Heart a D. However, SUS represents valid and reliable instrument that can be used with a small number of participants, but it is not recommended as a diagnostic tool to examine usability problems [19].

Second, also in the ISONORM score Valve Assist 2 shows better usability results. In contrast to SUS, ISONORM is a good choice to find out which dialogue principles of human-system interaction (ISO 9241-110) relate to the usability problem [19, 23, 25]. We found that Valve Assist 2 performed better regarding suitability for learning, suitability for individualization and self-descriptiveness than Structural Heart. In usability practice, a score ≥ 1 is a criterion that the minimum requirements for good ergonomics of human-system interaction (ISO 9241-110:2006) assessed in the ISONORM questionnaire have been met [23].

Overall, Valve Assist 2 met this criterion in contrast to Structural Heart. As a limitation it should be noticed, that Valve Assist 2 only met the good software criterion in dialogue principles in suitability for learning.

In addition, measurements with Valve Assist 2 were made with fewer questions asked to experts. Further, in T2, the beginners measured with Valve Assist 2 faster and even without any questions asked to the experts. The better results after training-free period underline the advantage of Valve Assist 2 in suitability for learning besides the ISONORM result.

When considering the number of correctly selected valves, the result is not entirely clear. On the one hand, more correct valve sizes were selected with Structural Heart in T1. On the other hand, beginners chose more often correct valve sizes with Valve Assist 2 in T2. In contrast to Valve Assist 2 (72.73% to 69.70%), the number of correctly selected valve sizes for Structural Heart (93.94% to 40.00%) decreased significantly from T1 to T2. These results may apply only to program-inexperienced users, as at least for Structural Heart, it was shown that the correct measurements are comparable to other programs [12]. Structural Heart’s lower result on T2, therefore stands also for usability problems especially in terms of suitability for learning.

Our study has some strengths and limitations. First, our study used a panel of 9 experts. Although we recruited all available TAVR experts from the Heart Center Brandenburg, experts from other TAVR centers should also be included in order to reach a panel size above 10 [31].

Second, true valve size in patient 2 can only be approximately determined because measurements by expert group are between 2 TAVR sizes. Besides the fact that correct measurement between 2 TAVR sizes is subject of current research [32], a ICC of 0.981 is considered as an excellent degree of interrater reliability [33]. Third, the training-free interval between T1 and T2 differed in the minimum and in the maximum (minimum 13 days, maximum 22 days). Nonetheless, we found no association between duration of the training-free days between T1 and T2 and correct measured valve size in T2. Fourth, 30 min of training before the first use of aortic annulus sizing software programs may be too short. However, all beginners were able to complete the measurements.

Fifth, if a beginner had to ask the expert for help with the next step, we recorded it, but did not consider the measurement as failed. Moderated usability tests may not be common, but for the complex TAVR planning, it is realistic in clinical practice to seek expert advice when needed.

Our study shows significant better usability results for Valve Assist 2 compared to Structural Heart in program-inexperienced users. This suggests that choosing a particular semi-automated TAVR planning software may have an impact on the TAVR planning process.

Further studies with a study population of experienced TAVR specialists are needed to assess the impact of usability of semi-automated software programs on sizing and eventually clinical outcomes.
